# Supplementary material for: A phenolic-rich extract from Ugni molinae berries reduces abnormal protein aggregation in a cellular model of Huntington’s disease
Source: PLoS One. 2021 Jul 29;16(7):e0254834. doi: 10.1371/journal.pone.0254834 (PMC8320977; doi:10.1371/journal.pone.0254834)
Supplement: S1 Table — SE = Selected extract; Rt = retention time. Semiquantification was carried out in respect to the higher normalized area obtained from selective ion mode in LC-MS analysis. n.i. = not identified. aCEPEDEQ library; bReSpect for Phytochemicals Database; cReference: Peña-Cerda et al., 2017 [24]; Wyrepkowski et al., 2014 [27]. (DOC) [file pone.0254834.s001.doc]

| **S1 Table.** Tentative identification and relative quantification of phenolic compounds in the SEs of *U. molinae* fruits from different genotypes | | | | | | | | | | | | |
| --- | --- | --- | --- | --- | --- | --- | --- | --- | --- | --- | --- | --- |
|  |  |  |  |  |  |  | **Semi-quantification to the highest normalized area (%)** | | | | |  |
| **#** | **Rt (min)** | **[M-H]^-^  (m/z)** | **Fragments (m/z)** | | | **Tentative Identification** | **14-4** | **19-1** | **19-1**  **ha** | **23-2** | **27-1** | **Refs** |
| 1 | 2.9 | 503 | 341 | 179 |  | Caffeic acid dihexoside | 75,1 | 100,0 | 99,1 | 52,9 | 26,9 | a,b |
| 2 | 5.2 | 633 | 587 | 301 |  | HHDP-galloyl hexose | 19,5 | 100,0 | 0,0 | 0,0 | 21,9 | a,c |
| 3 | 6.0 | 495 | 343 | 169 |  | Digalloylquinic acid | 2,2 | 35,2 | 100,0 | 30,5 | 75,0 | a,c |
| 4 | 6.3 | 483 | 331 | 233 | 169 | Digalloyl glucose | 0,0 | 100,0 | 0,0 | 0,0 | 0,0 | a |
| 5 | 6,3 | 633 | 421 | 301 |  | HHDP-galloyl hexose | 0,0 | 61,9 | 0,0 | 0,0 | 100,0 | a,c |
| 6 | 7.1 | 633 | 421 | 301 |  | HHDP-galloyl hexose | 100,0 | 0,0 | 0,0 | 0,0 | 0,0 | a,c |
| 7 | 7.5 | 495 | 343 | 169 |  | Digalloylquinic acid | 27,0 | 33,4 | 100,0 | 25,7 | 85,3 | a |
| 8 | 8.4 | 633 | 421 | 301 |  | HHDP-galloyl hexose | 0,0 | 100,0 | 72,8 | 0,0 | 72,8 | a |
| 9 | 8.6 | 577 | 425 | 451 |  | Procyanidin dimer B | 100,0 | 0,0 | 0,0 | 0,0 | 0,0 | a |
| 10 | 8.7 | 503 | 341 | 179 |  | Caffeic acid dihexoside | 0,0 | 100,0 | 0,0 | 0,0 | 0,0 | a |
| 11 | 8.9 | 495 | 343 | 169 |  | Digalloylquinic acid | 7,5 | 2,8 | 51,0 | 37,2 | 100,0 | a |
| 12 | 9.8 | 577 | 425 | 450 |  | Procyanidin dimer B | 100,0 | 0,0 | 0,0 | 0,0 | 0,0 | a |
| 13 | 10.5 | 483 | 423 | 271 | 169 | Digalloylglucose | 100,0 | 0,0 | 38,8 | 0,0 | 0,0 | a |
| 14 | 10.7 | 633 | 482 | 301 |  | HHDP-galloyl hexose | 100,0 | 0,0 | 0,0 | 0,0 | 0,0 | a |
| 15 | 10.8 | 495 | 343 | 169 |  | Digalloylquinic acid | 44,1 | 60,5 | 73,7 | 38,7 | 100,0 | a |
| 16 | 10.8 | 289 | 245 |  |  | (Epi)catechin | 100,0 | 0,0 | 0,0 | 0,0 | 0,0 | a,b |
| 17 | 12.2 | 289 | 245 |  |  | (Epi)catechin | 100,0 | 8,6 | 0,0 | 0,0 | 0,0 | a,b |
| 18 | 12.4 | 453 | 313 | 285 | 169 | Digalloylpentose | 0,0 | 0,0 | 100,0 | 0,0 | 0,0 | a |
| 19 | 13.0 | 625 | 463 | 301 |  | Quercetin-O-dihexoside | 100,0 | 0,0 | 0,0 | 0,0 | 0,0 | a |
| 20 | 13.1 | 325 | 163 | 145 |  | Unknown | 0,0 | 14,6 | 0,0 | 56,6 | 100,0 | n.i. |
| 21 | 13.1 | 431 | 269 |  |  | Apigenin-O-glucoside | 0,0 | 100,0 | 0,0 | 0,0 | 0,0 | a |
| 22 | 13.8 | 635 | 465 | 283 |  | Trigalloyl glucose | 0,0 | 94,7 | 100,0 | 0,0 | 0,0 | a |
| 23 | 14.2 | 631 | 479 | 317 |  | Myricetin-O-galloyl hexoside | 10,2 | 39,6 | 100,0 | 56,1 | 34,6 | a |
| 24 | 15.0 | 454 | 313 | 169 |  | Digalloylpentose | 100,0 | 78,2 | 0,0 | 0,0 | 0,0 | a |
| 25 | 15.4 | 631 | 479 | 317 |  | Myricetin-O-galloyl hexoside | 9,1 | 32,3 | 100,0 | 36,5 | 36,7 | a |
| 26 | 15.6 | 635 | 465 | 271 |  | Trigalloyl glucose | 0,0 | 100,0 | 0,0 | 0,0 | 0,0 | a |
| 27 | 15.7 | 479 | 317 |  |  | Myricetin-O-hexoside | 100,0 | 0,0 | 0,0 | 0,0 | 0,0 | a |
| 28 | 15.7 | 448 | 285 |  |  | Luteolin-O-hexoside/Kaempferol-O-hexoside | 0,0 | 27,9 | 0,0 | 100,0 | 0,0 | a,b |
| 29 | 15.9 | 402 | 269 | 131 |  | Benzyl alcohol hexose pentose | 17,5 | 0,0 | 100,0 | 69,4 | 0,0 | a |
| 30 | 15.9 | 577 | 425 |  |  | Procyanidin dimer B | 100,0 | 0,0 | 0,0 | 0,0 | 0,0 | a |
| 31 | 16.1 | 453 | 313 |  |  | Digalloylpentose | 0,0 | 0,0 | 100,0 | 0,0 | 0,0 | a |
| 32 | 16.2 | 479 | 317 |  |  | Myricetin-O-hexoside | 89,3 | 24,7 | 65,1 | 100,0 | 84,5 | a |
| 33 | 16.6 | 445 | 448 | 285 |  | Kaempferol-O-hexoside | 0,0 | 0,0 | 100,0 | 0,0 | 0,0 | a |
| 34 | 17.0 | 615 | 463 | 313 |  | Quercetin-O-galloyl hexoside | 0,0 | 31,9 | 100,0 | 0,0 | 47,4 | a |
| 35 | 17.0 | 577 | 425 |  |  | Procyanidin dimer B | 100,0 | 0,0 | 0,0 | 0,0 | 0,0 | a |
| 36 | 17.0 | 631 | 479 | 317 |  | Myricetin-O-galloyl hexoside | 6,8 | 100,0 | 58,7 | 38,4 | 77,4 | a |
| 37 | 17.3 | 477 | 301 |  |  | Quercetin-O-glucuronide | 0,0 | 100,0 | 0,0 | 0,0 | 0,0 | a |
| 38 | 17.6 | 449 | 316 |  |  | Myricetin-O-pentoside | 21,5 | 100,0 | 0,0 | 0,0 | 5,8 | a |
| 39 | 17.7 | 463 | 301 |  |  | Quercetin-O-hexoside | 34,7 | 66,5 | 100,0 | 82,3 | 91,2 | a |
| 40 | 18.3 | 755 | 453 | 291 |  | Unknown | 73,8 | 0,0 | 0,0 | 100,0 | 13,2 | n.i. |
| 41 | 18.5 | 479 | 317 |  |  | Myricetin-O-hexoside | 10,1 | 1,0 | 73,4 | 100,0 | 62,8 | a |
| 42 | 18.7 | 433 | 301 | 170 |  | Quercetin-O-pentoside | 100,0 | 0,0 | 0,0 | 0,0 | 0,0 | a |
| 43 | 19.0 | 625 | 463 | 301 |  | Quercetin-O-dihexoside | 66,7 | 0,0 | 0,0 | 0,0 | 100,0 | a |
| 44 | 19.1 | 461 | 317 |  |  | Myricetin-O-deoxyhexoside | 30,9 | 0,0 | 0,0 | 100,0 | 0,0 | a |
| 45 | 19.1 | 615 | 463 | 301 |  | Quercetin-O-galloyl hexoside | 0,0 | 0,0 | 100,0 | 0,0 | 43,4 | a |
| 46 | 19.7 | 479 | 316 | 179 |  | Myricetin-O-hexoside | 22,1 | 32,8 | 100,0 | 94,1 | 82,6 | a |
| 47 | 20.0 | 615 | 463 | 301 |  | Quercetin-O-galloyl hexoside | 19,7 | 11,8 | 100,0 | 72,8 | 41,2 | a |
| 48 | 20.4 | 625 | 463 | 301 |  | Quercetin-O-dihexoside | 31,9 | 0,0 | 100,0 | 71,6 | 0,0 | a |
| 49 | 20.7 | 449 | 317 |  |  | Myricetin-O-pentoside | 0,0 | 81,1 | 0,0 | 100,0 | 26,2 | a |
| 50 | 20.7 | 441 | 289 | 169 |  | (Epi)catechin-O-gallate | 100,0 | 0,0 | 0,0 | 0,0 | 0,0 | a,b |
| 51 | 20.8 | 433 | 301 |  |  | Quercetin-O-pentoside | 90,6 | 24,1 | 100,0 | 86,0 | 71,9 | a |
| 52 | 21.0 | 615 | 463 | 301 |  | Quercetin-O-galloyl hexoside | 0,0 | 100,0 | 0,0 | 0,0 | 0,0 | a |
| 53 | 21.1 | 625 | 463 | 301 |  | Quercetin-O-dihexoside | 0,0 | 0,0 | 100,0 | 0,0 | 0,0 | a |
| 54 | 21.2 | 463 | 316 |  |  | Myricetin-O-rhamnoside | 0,0 | 6,0 | 86,7 | 100,0 | 78,1 | a |
| 55 | 21.5 | 449 | 316 |  |  | Myricetin-O-pentoside | 0,0 | 100,0 | 26,0 | 0,0 | 0,0 | a |
| 56 | 21.5 | 477 | 315 |  |  | Isorhamnetin-O-hexoside | 0,0 | 100,0 | 0,0 | 0,0 | 0,0 | a |
| 57 | 21.7 | 433 | 300 |  |  | Quercetin-O-pentoside | 17,9 | 100,0 | 0,0 | 0,0 | 0,0 | a |
| 58 | 22.0 | 477 | 301 |  |  | Quercetin-O-glucuronide | 19,2 | 0,0 | 100,0 | 28,2 | 36,7 | a |
| 59 | 22.6 | 463 | 301 |  |  | Quercetin-O-hexoside | 100,0 | 16,6 | 58,3 | 73,3 | 39,4 | a |
| 60 | 22.6 | 301 | 301 | 257 | 178 | Ellagic acid | 0,0 | 0,0 | 0,0 | 0,0 | 100,0 | a,c |
| 61 | 23.0 | 477 | 301 |  |  | Quercetin-O-glucuronide | 49,2 | 1,2 | 98,6 | 0,0 | 100,0 | a |
| 62 | 23.1 | 463 | 301 |  |  | Quercetin-O-hexoside | 0,0 | 0,0 | 0,0 | 0,0 | 100,0 | a |
| 63 | 23.6 | 625 | 463 | 301 |  | Quercetin-O-dihexoside | 100,0 | 0,0 | 0,0 | 0,0 | 0,0 | a |
| 64 | 24.2 | 599 | 463 | 301 |  | Quercetin-hexose-protocatechuic acid | 19,3 | 31,0 | 30,9 | 100,0 | 54,2 | a |
| 65 | 24.6 | 447 | 284 | 300 | 327 | Kaempferol-O-hexoside | 0,0 | 4,2 | 100,0 | 0,0 | 6,5 | a |
| 66 | 24.7 | 433 | 301 |  |  | Quercetin-O-pentoside | 0,0 | 100,0 | 4,4 | 18,9 | 15,8 | a |
| 67 | 25.1 | 447 | 301 |  |  | Quercetin-O-rhamnoside | 0,0 | 0,0 | 0,0 | 100,0 | 0,0 | a |
| 68 | 25.5 | 463 | 301 | 255 | 169 | Quercetin-O-hexoside | 0,0 | 0,0 | 0,0 | 0,0 | 100,0 | a |
| 69 | 25.6 | 477 | 315 |  |  | Isorhamnetin-O-hexoside | 0,0 | 83,0 | 100,0 | 35,7 | 0,0 | a |
| 70 | 26.0 | 505 | 463 | 301 |  | Quercetin-O-acetyl hexoside | 19,2 | 0,0 | 40,9 | 100,0 | 0,0 | a |
| 71 | 26.0 | 461 | 285 |  |  | Kaempferol-O-glucuronide | 0,0 | 0,0 | 0,0 | 100,0 | 0,0 | a |
| 72 | 26.2 | 447 | 301 | 284 | 179 | Quercetin-O-rhamnoside | 7,9 | 0,0 | 19,9 | 100,0 | 30,8 | a |
| 73 | 26.5 | 475 | 301 |  |  | Luteolin-methyl ether-O-glucuronide | 0,0 | 0,0 | 0,0 | 100,0 | 0,0 | a |
| 74 | 26.7 | 463 | 301 |  |  | Quercetin-O-hexoside | 0,0 | 0,0 | 0,0 | 100,0 | 82,0 | a |
| 75 | 26.7 | 477 | 314 | 301 |  | Isorhamnetin-O-hexoside | 33,7 | 0,0 | 0,0 | 0,0 | 100,0 | a |
| 76 | 27.1 | 613 | 317 | 463 |  | Myricetin-O-galloyl deoxyhexoside | 0,0 | 0,0 | 0,0 | 100,0 | 0,0 | a |
| 77 | 27.2 | 417 | 284 |  |  | Kaempferol-O-pentoside | 0,0 | 100,0 | 0,0 | 0,0 | 0,0 | a |
| 78 | 27.4 | 505 | 301 | 463 | 179 | Quercetin-O-acetyl hexoside | 38,3 | 2,9 | 63,4 | 78,2 | 100,0 | a |
| 79 | 27.9 | 625 | 463 | 301 |  | Quercetin-O-dihexoside | 0,0 | 100,0 | 0,0 | 27,8 | 38,2 | a |
| 80 | 27.9 | 479 | 317 |  |  | Myricetin-O-hexoside | 0,0 | 0,0 | 79,9 | 100,0 | 53,2 | a |
| 81 | 28.0 | 463 | 301 |  |  | Quercetin-O-hexoside | 0,0 | 0,0 | 0,0 | 100,0 | 0,0 | a |
| 82 | 28.2 | 315 | 300 | 179 | 107 | Isorhamnetin | 100,0 | 0,0 | 0,0 | 0,0 | 0,0 | a |
| 83 | 28.3 | 639 | 493 | 331 |  | Methoxyquercetin-O-hexoside | 0,0 | 55,2 | 0,0 | 0,0 | 100,0 | a |
| 84 | 28.4 | 417 | 285 |  |  | Kaempferol-O-pentoside | 0,0 | 100,0 | 0,0 | 0,0 | 0,0 | a |
| 85 | 28.5 | 476 | 300 |  |  | Luteolin-methyl ether-O-glucuronide | 0,0 | 100,0 | 0,0 | 0,0 | 0,0 | a |
| 86 | 28.5 | 505 | 487 | 301 |  | Quercetin-O-acetyl hexoside | 3,8 | 1,0 | 70,8 | 100,0 | 50,2 | a |
| 87 | 28.6 | 301 | 300 |  |  | Ellagic acid | 0,0 | 0,0 | 0,0 | 0,0 | 100,0 | a,c |
| 88 | 28.9 | 431 | 285 |  |  | Kaempferol-O-rhamnoside | 0,0 | 0,0 | 100,0 | 0,0 | 0,0 | a |
| 89 | 29.4 | 490 | 447 | 315 | 284 | Isorhamnetin-O-glucuronide | 0,0 | 0,0 | 100,0 | 0,0 | 0,0 | a |
| 90 | 29.6 | 645 | 479 | 317 |  | Galloylmethylmyricetin hexoside | 0,0 | 29,5 | 100,0 | 0,0 | 18,0 | a |
| 91 | 30.2 | 610 | 463 | 301 |  | Quercetin-O-rhamnosylhexoside | 0,0 | 100,0 | 77,2 | 40,7 | 48,2 | a |
| 92 | 30.6 | 639 | 463 | 301 |  | Isorhamnetin-O-dihexoside | 0,0 | 0,0 | 100,0 | 72,0 | 50,4 | a |
| 93 | 31.2 | 489 | 285 | 227 |  | Kaempferol-O-acetyl hexoside | 0,0 | 0,0 | 100,0 | 0,0 | 47,6 | a |
| 94 | 31.4 | 607 | 463 | 301 |  | Quercetin-O-rhamnosyl hexoside | 100,0 | 0,0 | 0,0 | 0,0 | 0,0 | a |
| 95 | 31.5 | 645 | 479 | 316 |  | Galloylmethylmyricetin hexoside | 0,0 | 0,0 | 100,0 | 30,9 | 0,0 | a |
| 96 | 31.8 | 609 | 464 | 301 |  | Quercetin-O-rhamnosyl-O-hexoside | 0,0 | 0,0 | 0,0 | 53,6 | 100,0 | a |
| 97 | 32.0 | 593 | 447 | 285 |  | Kaempferol-O-coumaroyl glucoside | 0,0 | 0,0 | 100,0 | 40,5 | 0,0 | a |
| 98 | 32.4 | 301 | 179 | 151 |  | Quercetin | 100,0 | 19,2 | 99,5 | 63,6 | 46,7 | a,b,c |
| 99 | 32.4 | 331 | 316 |  |  | Myricetin methyl ether | 0,0 | 0,0 | 100,0 | 40,2 | 5,8 | a |
| 100 | 32.7 | 623 | 477 | 315 |  | Isorhamnetin-O-rutinoside | 0,0 | 100,0 | 0,0 | 0,0 | 26,3 | a |
| 101 | 33.6 | 595 | 579 | 287 |  | Eriodictyol-O-rutinoside | 0,0 | 0,0 | 100,0 | 0,0 | 0,0 | a |
| 102 | 36.0 | 285 | 285 |  |  | Kaempferol | 0,0 | 7,4 | 100,0 | 3,4 | 8,6 | a,b |
| 103 | 36.4 | 315 | 300 |  |  | Isorhamnetin | 0,0 | 16,2 | 29,0 | 0,0 | 100,0 | a |
| 104 | 36.8 | 462 | 301 | 181 |  | Quercetin-O-hexoside | 0,0 | 0,0 | 0,0 | 0,0 | 100,0 | a |

SE= Selected extract; Rt = retention time. Semiquantification was carried out in respect to the higher normalized area obtained from selective ion mode in LC-MS analysis. n.i. = not identified. ^a^CEPEDEQ library; ^b^ReSpect for Phytochemicals Database; ^c^Reference: Peña-Cerda et al., 2017^24^; Wyrepkowski et al., 2014^27^
