# Supplementary material for: A phenolic-rich extract from Ugni molinae berries reduces abnormal protein aggregation in a cellular model of Huntington’s disease
Source: PLoS One. 2021 Jul 29;16(7):e0254834. doi: 10.1371/journal.pone.0254834 (PMC8320977; doi:10.1371/journal.pone.0254834)
Supplement: S1 Fig — To analyze the correlation between the TPC of the SEs and their effects over intracellular polyQ79-EGFP inclusions a linear regression between both sets of data was performed. (DOC) [file pone.0254834.s002.doc]

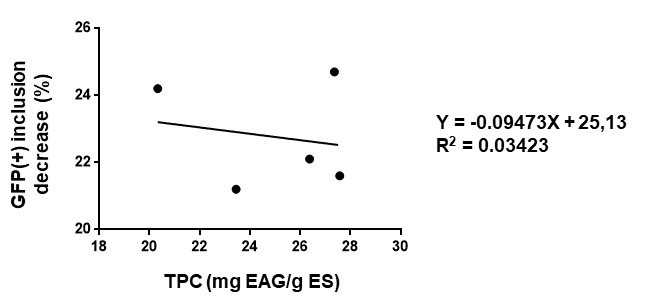


**S1 Fig. The TPC is not correlated to the effect of the SEs on the number of polyQ_79_-EGFP intracellular inclusions.** To analyze the correlation between the TPC of the SEs and their effects over intracellular polyQ_79_-EGFP inclusions a linear regression between both sets of data was performed.
