# Supplementary material for: A phenolic-rich extract from Ugni molinae berries reduces abnormal protein aggregation in a cellular model of Huntington’s disease
Source: PLoS One. 2021 Jul 29;16(7):e0254834. doi: 10.1371/journal.pone.0254834 (PMC8320977; doi:10.1371/journal.pone.0254834)
Supplement: S2 Fig — (A) TPC of ETE 19–1 was assessed by FC method to check for major changes in its phenolic composition during time. (B) HEK293 cells were seeded in 6-well plate and treatment with ETE 19–1 was performed at two concentrations (100 and 200 µg/mL) using quercetin (10 µM) and DMSO as controls. Cells were stained with 0.4% trypan blue and manually counted in duplicates using a Neubauer improved chamber at 0, 24, 48, 72, and 96 h. (C) HEK293 cells were seeded in 96-well plates (1x10^4^ cells per well) and treated as previously described for 24 and 48 h. Cell number was indirectly determined by the MTS assay. (D) HEK293 cells were treated with 100 or 200 µg/ml of ETE 19–1, 10 µM Quercetin, vehicle (DMSO) or left untreated (NT) for 16 h. DNA was stained with 5 µg/ml of propidium iodide (PI) and cells were analyzed by FACS. (E) HEK293 cells were pre-treated with 100 or 200 µg/ml ETE 19–1, 10 µM Quercetin or vehicle (DMSO) for 16 h and then treated with 500 µM H2O2 for 2 h. Culture media was replaced with a solution containing 10 µM DHE in HBSS, incubated 20 min at 37°C, and protected from light. Then, cells were washed in HBSS, trypsinized, resuspended in 200 µL HBSS, and the DHE fluorescence was determined by FACS. Results are reported as mean ± SD of three independent measurements for spectrophotometric analysis, and as mean ± SEM for cell-based studies. Statistical analyses were carried out using Graph-Pad Prism 6.0 software. T-test, one-way ANOVA and Tukey’s or Dunnet’s multiple comparison test were used to analyze the data, considering p ≤ 0.05 as significant. NT = not treated; DMSO = dimethyl sulfoxide; GAE = gallic acid equivalents; DE = Dry extract; DHE = Dihydroethidium; MFI = mean fluorescence intensity. (DOC) [file pone.0254834.s003.doc]

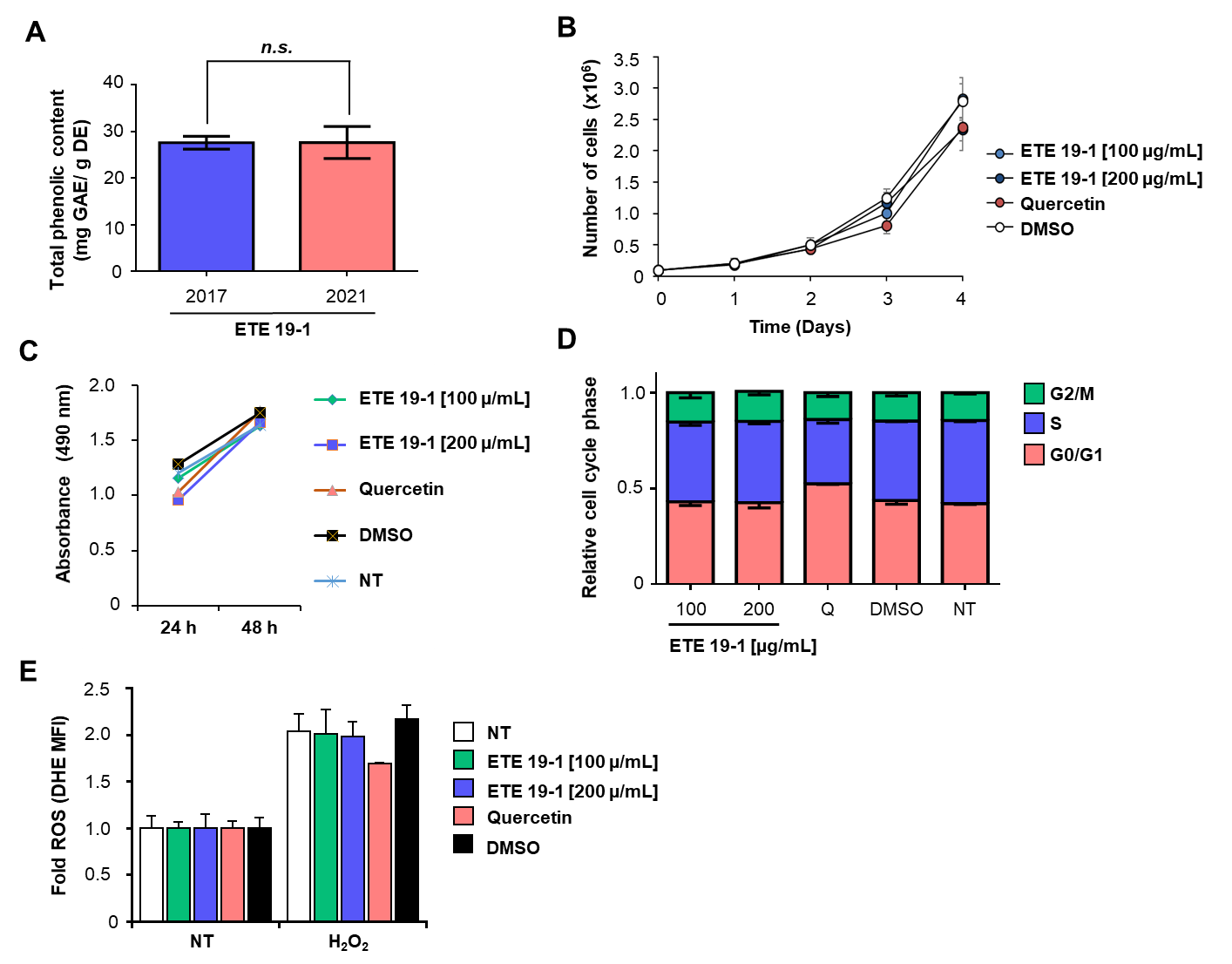


**S2 Fig.** **ETE 19-1 treatment does not affect cell proliferation, cell cycle, or induce changes in ROS levels.** (A) TPC of ETE 19-1 was assessed by FC method to check for major changes in its phenolic composition during time. (B) HEK293 cells were seeded in 6-well plate and treatment with ETE 19-1 was performed at two concentrations (100 and 200 µg/mL) using quercetin (10 µM) and DMSO as controls. Cells were stained with 0.4% trypan blue and manually counted in duplicates using a Neubauer improved chamber at 0, 24, 48, 72, and 96 h. (C) HEK293 cells were seeded in 96-well plates (1x10^4^ cells per well) and treated as previously described for 24 and 48 h. Cell number was indirectly determined by the MTS assay. (D) HEK293 cells were treated with 100 or 200 µg/ml of ETE 19-1, 10 µM Quercetin, vehicle (DMSO) or left untreated (NT) for 16 h. DNA was stained with 5 µg/ml of propidium iodide (PI) and cells were analyzed by FACS. (E) HEK293 cells were pre-treated with 100 or 200 µg/ml ETE 19-1, 10 µM Quercetin or vehicle (DMSO) for 16 h and then treated with 500 µM H_2_O_2_ for 2 h. Culture media was replaced with a solution containing 10 µM DHE in HBSS, incubated 20 min at 37°C, and protected from light. Then, cells were washed in HBSS, trypsinized, resuspended in 200 µL HBSS, and the DHE fluorescence was determined by FACS. Results are reported as mean ± SD of three independent measurements for spectrophotometric analysis, and as mean ± SEM for cell-based studies. Statistical analyses were carried out using Graph-Pad Prism 6.0 software. T-test, one-way ANOVA and Tukey’s or Dunnet’s multiple comparison test were used to analyze the data, considering p ≤ 0.05 as significant. NT = not treated; DMSO = dimethyl sulfoxide; GAE = gallic acid equivalents; DE = Dry extract; DHE = Dihydroethidium; MFI = mean fluorescence intensity.
