# Supplementary material for: A phenolic-rich extract from Ugni molinae berries reduces abnormal protein aggregation in a cellular model of Huntington’s disease
Source: PLoS One. 2021 Jul 29;16(7):e0254834. doi: 10.1371/journal.pone.0254834 (PMC8320977; doi:10.1371/journal.pone.0254834)
Supplement: S3 Fig — (A) The effect of ETE 19–1 treatment over pCHK1 expression was analyzed by Western Blot. HEK293 cells were seeded in 6-well plate and treatment with ETE 19–1 (100 and 200 µg/mL) for 16 h. Quercetin, DMSO and etoposide (Eto) were used as controls. After 16 h, cells were collected, and total proteins extracted. pCHK1 levels was analyzed using an Anti-pCHK1 antibody. HSP90 expression was monitored as loading control. (B) The effect of ETE 19–1 treatment over p21 was analyzed by Quantitative real-time PCR. HEK293 cells were seeded in 6-well plate and treatment with ETE 19–1 (100 and 200 µg/mL) for 16 h. Quercetin, DMSO and etoposide (Eto) were used as controls. Total RNA was prepared from HEK293 cells using Trizol and cDNA was synthesized with SuperScript III (Invitrogen) using random primers p(dN)6. Quantitative real-time PCR reactions were performed using SYBRgreen fluorescent reagent and/or EvaGreenTM using a Stratagene Mx3000P system. The relative amounts of human p21 mRNAs were calculated from the values of comparative threshold cycle by using human GAPDH as a Housekeeping. Results are reported as mean ± SEM of three independent measurements. Statistical analyses were carried out using Graph-Pad Prism 6.0 software. One-way ANOVA and Dunnet’s multiple comparison test were used to analyze the data, considering p ≤ 0.05 as significant. NT = not treated; DMSO = dimethyl sulfoxide; Q = Quercetin; Eto = Etoposide. (DOC) [file pone.0254834.s004.doc]

**
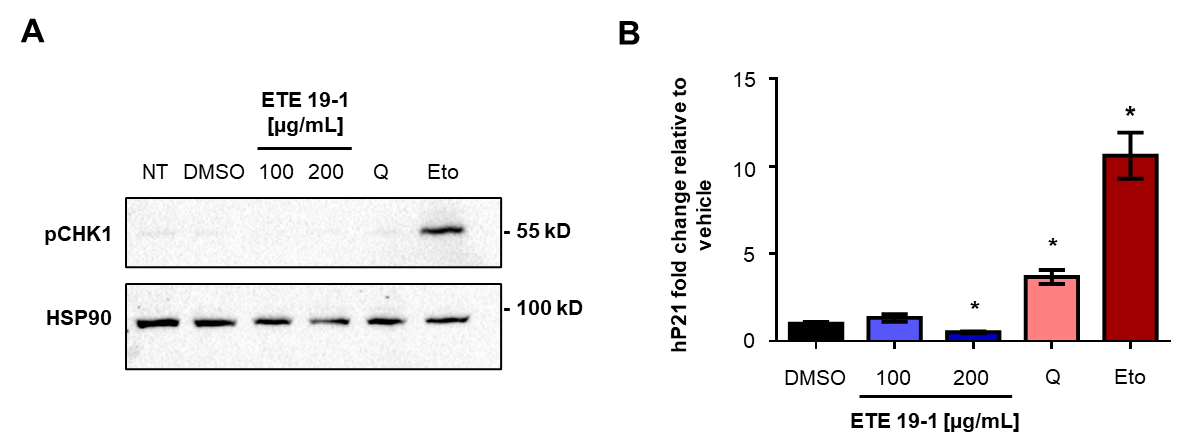
**

**S3 Fig.** **ETE 19-1 treatment does not induce DNA damage.** (A) The effect of ETE 19-1 treatment over phosphorylated CHK1 (pCHK1) expression was analyzed by Western Blot. HEK293 cells were seeded in 6-well plate and treatment with ETE 19-1 (100 and 200 µg/mL) for 16 h. Quercetin, DMSO and 25 μM etoposide (Eto) were used as controls. After 16 h, cells were collected, and total proteins extracted. pCHK1 levels was analyzed using an Anti-pCHK1 antibody. HSP90 expression was monitored as loading control. (B) The effect of ETE 19-1 treatment over p21 was analyzed by quantitative real-time PCR. HEK293 cells were seeded in 6-well plate and treatment with ETE 19-1 (100 and 200 µg/mL) for 16 h. Quercetin, DMSO and 25 μM etoposide (Eto) were used as controls. Total RNA was prepared from HEK293 cells using Trizol and cDNA was synthesized with SuperScript III (Invitrogen) using random primers p(dN)6. Quantitative real-time PCR reactions were performed using SYBRgreen fluorescent reagent and/or EvaGreenTM using a Stratagene Mx3000P system. The relative amounts of human p21 mRNAs were calculated from the values of comparative threshold cycle by using human GAPDH as a Housekeeping. Results are reported as mean ± SEM of three independent measurements. Statistical analyses were carried out using Graph-Pad Prism 6.0 software. One-way ANOVA and Dunnet’s multiple comparison test were used to analyze the data, considering p ≤ 0.05 as significant. NT = not treated; DMSO = dimethyl sulfoxide; Q = Quercetin; Eto = Etoposide.
