# Supplementary material for: A phenolic-rich extract from Ugni molinae berries reduces abnormal protein aggregation in a cellular model of Huntington’s disease
Source: PLoS One. 2021 Jul 29;16(7):e0254834. doi: 10.1371/journal.pone.0254834 (PMC8320977; doi:10.1371/journal.pone.0254834)
Supplement: S4 Fig — Senescence was induced in IMR90 cells as described in the methods section. Senescent cells were treated with ETE 19–1 and 3 concentrations were tested (50, 100, and 200 µg/mL). (A) The presence of cleaved caspase 3 was evaluated by immunofluorescence at 0, 24, and 48 hrs. 100 cells were counted and ABT 263 was used as a control. Cell nuclei can be seen in blue (Hoetsch) and cleaved caspase 3 can be seen in green. (B) Quantification of cleaved caspase 3 positive cells. Results are the mean of 2 experiments. (DOC) [file pone.0254834.s005.doc]

**
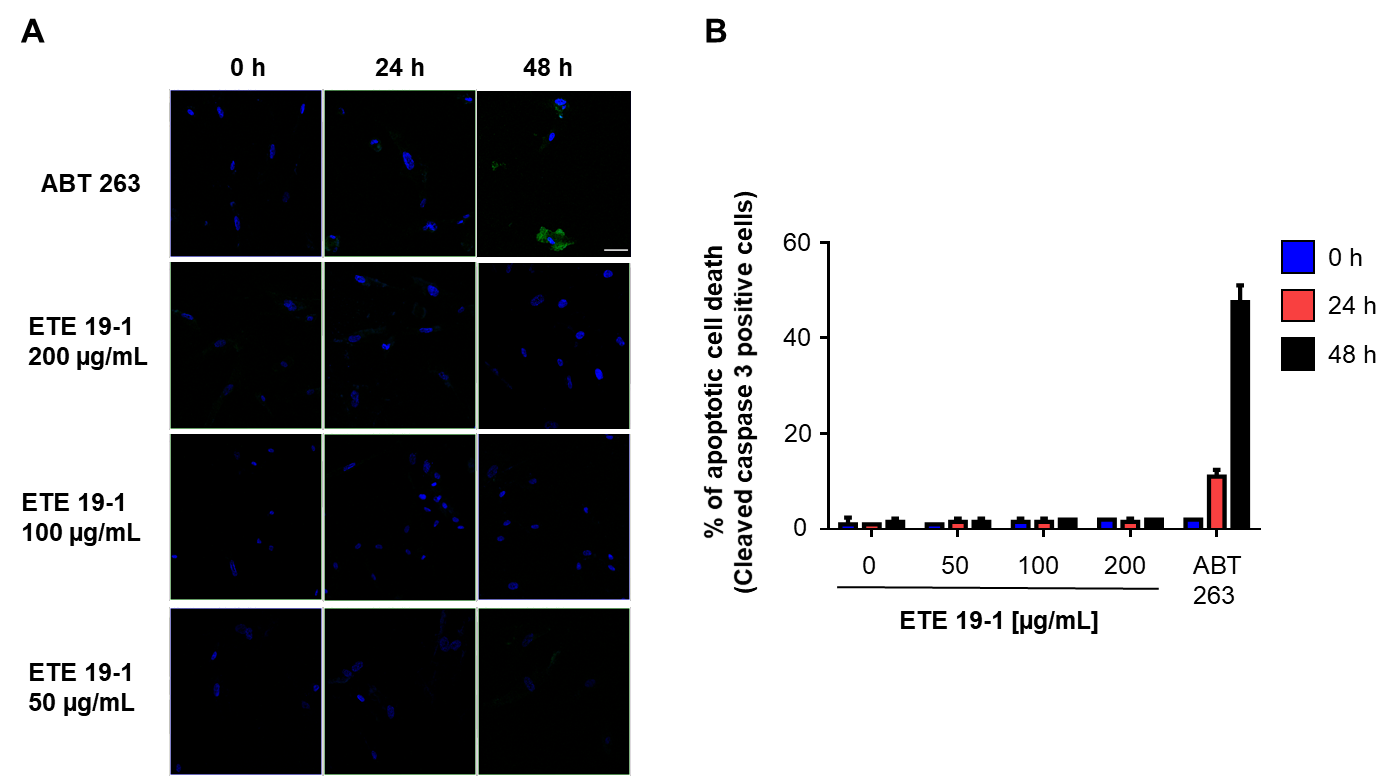
**

**S4 Fig.** **ETE 19-1 treatment does not induce death by apoptosis in senescent cells.** Senescence was induced in IMR90 cells as described in the methods section. Senescent cells were treated with ETE 19-1 and 3 concentrations were tested (50, 100, and 200 µg/mL). (**A)** The presence of cleaved caspase 3 was evaluated by immunofluorescence at 0, 24, and 48 hrs. 100 cells were counted and ABT 263 (senolytic) was used as positive control. Cell nuclei can be seen in blue (Hoetsch) and cleaved caspase 3 can be seen in green. **(B)** Quantification of cleaved caspase 3 positive cells. Results are the mean of 2 experiments.
