# Supplementary material for: A phenolic-rich extract from Ugni molinae berries reduces abnormal protein aggregation in a cellular model of Huntington’s disease
Source: PLoS One. 2021 Jul 29;16(7):e0254834. doi: 10.1371/journal.pone.0254834 (PMC8320977; doi:10.1371/journal.pone.0254834)
Supplement: Attachment — Submitted filename: PONE-D-21-01842 - Response to editor.doc [file pone.0254834.s007.doc]

**Branislav T. Šiler, Ph.D.**

**Academic Editor PLOS ONE**

**Re:** PONE-D-21-01842

Dear Dr. Šiler,

Thank you very much for your comments on our response to the reviewers. We have revised and addressed your comments to improve typographic and grammar errors in our manuscript.

As requested, changes were made in the following issues:

**L130** and elsewhere: Please use 1×, not 1X.

**L131** and further: CO2, O2, CaCl2 - "2" should stand in subscript.

**L199**, L216: Please do not start a sentence with a number. Rephrasing is needed. Use "×" instead of "x".

**L249** and elswhere: Please avoid italicizing expressions which do not need it. For instance, "ReSpect" is stated in both Roman and cursive.

**Figure 6:** "Pentoside" and "Hexoside" should not be capitalized.

**Main title:** "from" should not stand italicized.

**L36:** Please introduce the common name "murtilla" since used further in the manuscript.

**L38** and further: Once introduced in full, species' Latin name should be abbreviated as *U. molinae*

We have double checked our manuscript and we hope that this version will meet PLOS ONE’s publication criteria. We would also like to acknowledge your time and effort in handling this manuscript, and we hope that you will find that the revised manuscript deserves publication in PLOS ONE.

Please, do not hesitate to contact us if additional information is required.

Best regards,

Carla Delporte, Ph.D.

Corresponding Author

Claudio Hetz, Ph.D.

Corresponding Author
